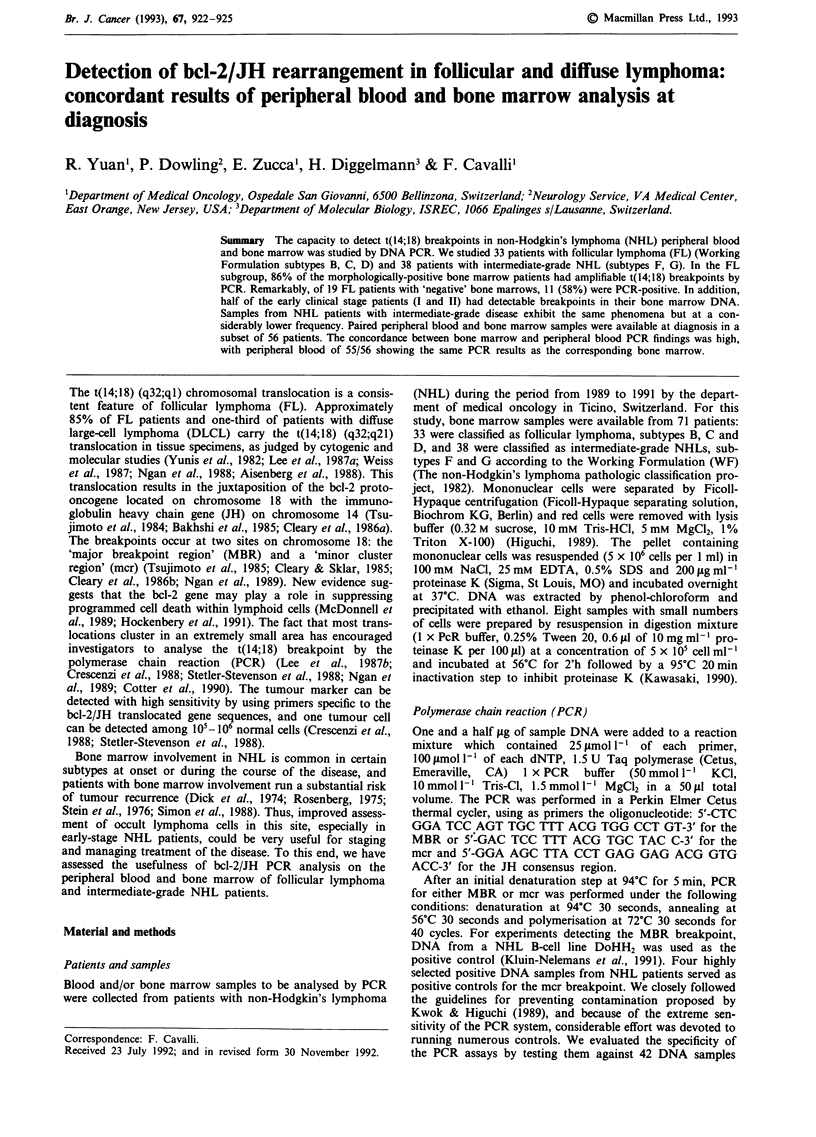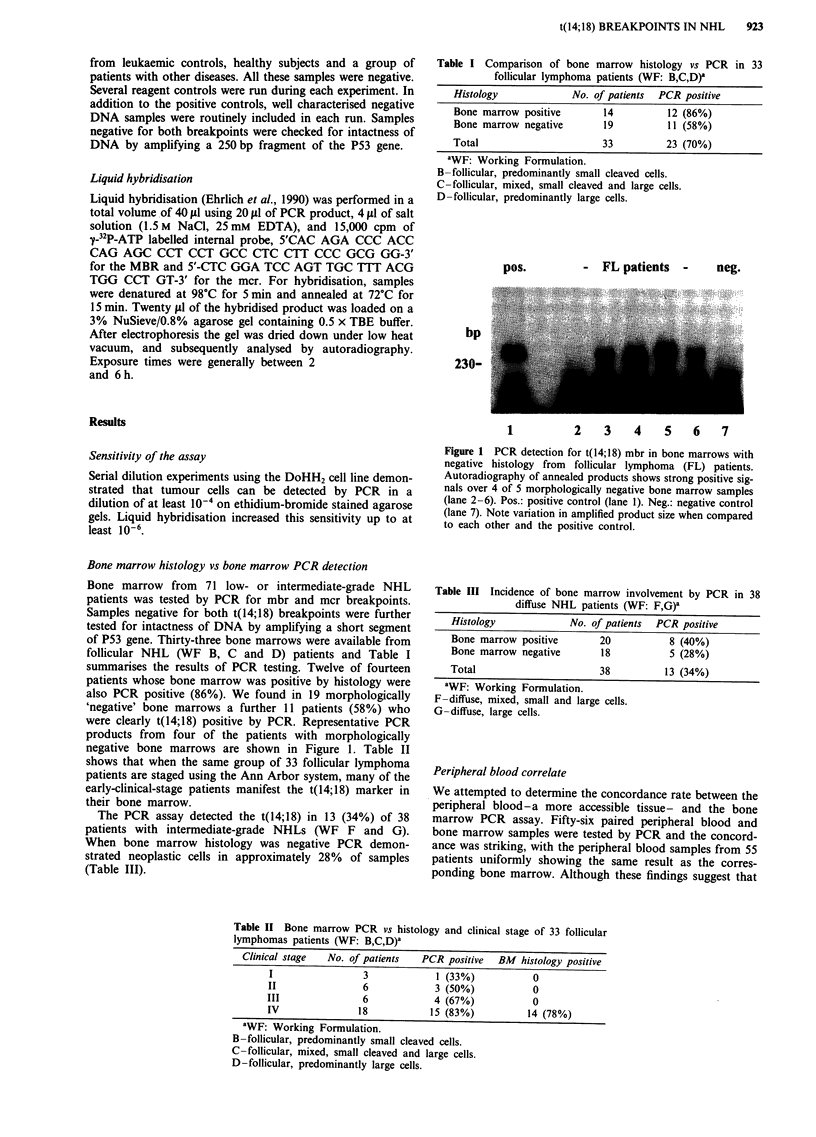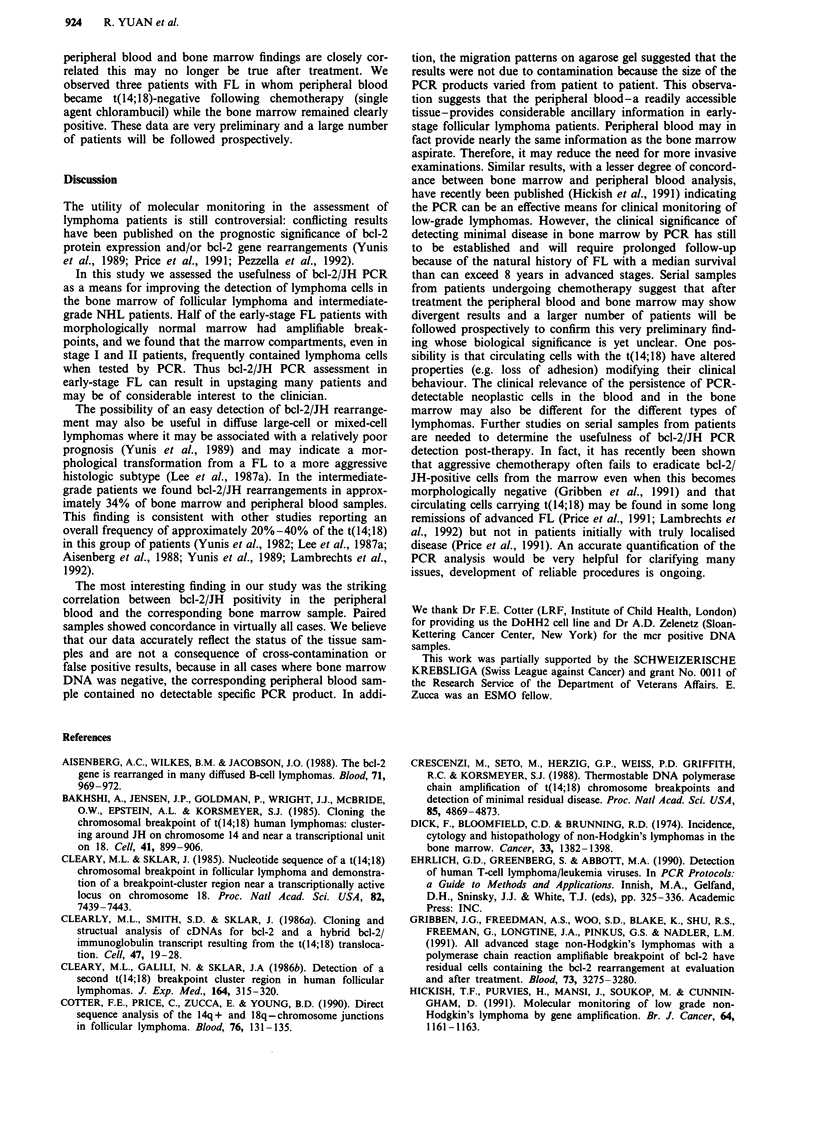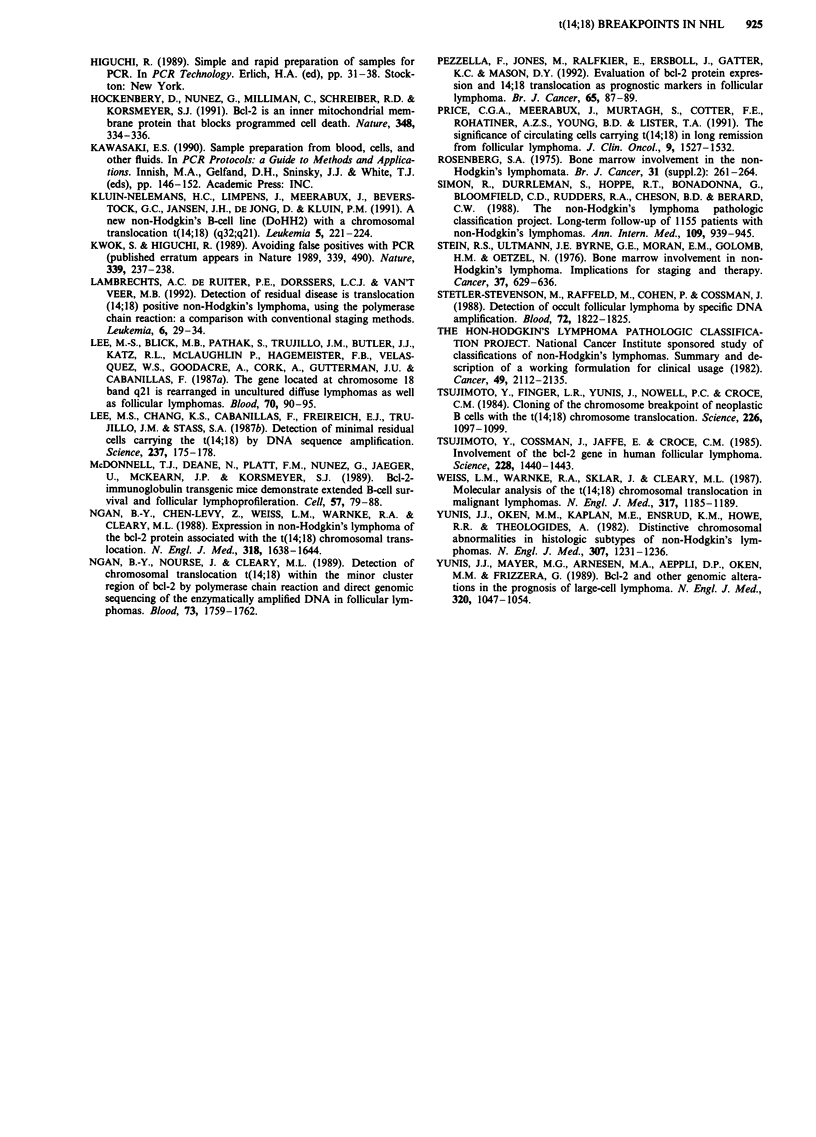# Detection of bcl-2/JH rearrangement in follicular and diffuse lymphoma: concordant results of peripheral blood and bone marrow analysis at diagnosis.

**DOI:** 10.1038/bjc.1993.171

**Published:** 1993-05

**Authors:** R. Yuan, P. Dowling, E. Zucca, H. Diggelmann, F. Cavalli

**Affiliations:** Department of Medical Oncology, Ospedale San Giovanni, Bellinzona, Switzerland.

## Abstract

**Images:**


Br. J. Cancer (1993), 67, 922–925 © Macmillan Press Ltd., 1993

### Detection of bcl-2/JH rearrangement in follicular and diffuse lymphoma: concordant results of peripheral blood and bone marrow analysis at diagnosis

R. Yuan[1], P. Dowling[2], E. Zucca[1], H. Diggelmann[3] & F. Cavalli[1]

[1]*Department of Medical Oncology, Ospedale San Giovanni, 6500 Bellinzona, Switzerland;* [2]*Neurology Service, VA Medical Center, East Orange, New Jersey, USA;* [3]*Department of Molecular Biology, ISREC, 1066 Epalinges s/Lausanne, Switzerland.*

**Summary** The capacity to detect t(14;18) breakpoints in non-Hodgkin's lymphoma (NHL) peripheral blood and bone marrow was studied by DNA PCR. We studied 33 patients with follicular lymphoma (FL) (Working Formulation subtypes B, C, D) and 38 patients with intermediate-grade NHL (subtypes F, G). In the FL subgroup, 86% of the morphologically-positive bone marrow patients had amplifiable t(14;18) breakpoints by PCR. Remarkably, of 19 FL patients with 'negative' bone marrows, 11 (58%) were PCR-positive. In addition, half of the early clinical stage patients (I and II) had detectable breakpoints in their bone marrow DNA. Samples from NHL patients with intermediate-grade disease exhibit the same phenomena but at a considerably lower frequency. Paired peripheral blood and bone marrow samples were available at diagnosis in a subset of 56 patients. The concordance between bone marrow and peripheral blood PCR findings was high, with peripheral blood of 55/56 showing the same PCR results as the corresponding bone marrow.

The t(14;18) (q32;q1) chromosomal translocation is a consistent feature of follicular lymphoma (FL). Approximately 85% of FL patients and one-third of patients with diffuse large-cell lymphoma (DLCL) carry the t(14;18) (q32;q21) translocation in tissue specimens, as judged by cytogenic and molecular studies (Yunis *et al.*, 1982; Lee *et al.*, 1987*a*; Weiss *et al.*, 1987; Ngan *et al.*, 1988; Aisenberg *et al.*, 1988). This translocation results in the juxtaposition of the bcl-2 proto-oncogene located on chromosome 18 with the immunoglobulin heavy chain gene (JH) on chromosome 14 (Tsujimoto *et al.*, 1984; Bakhshi *et al.*, 1985; Cleary *et al.*, 1986*a*). The breakpoints occur at two sites on chromosome 18: the 'major breakpoint region' (MBR) and a 'minor cluster region' (mcr) (Tsujimoto *et al.*, 1985; Cleary & Sklar, 1985; Cleary *et al.*, 1986b; Ngan *et al.*, 1989). New evidence suggests that the bcl-2 gene may play a role in suppressing programmed cell death within lymphoid cells (McDonnell *et al.*, 1989; Hockenbery *et al.*, 1991). The fact that most translocations cluster in an extremely small area has encouraged investigators to analyse the t(14;18) breakpoint by the polymerase chain reaction (PCR) (Lee *et al.*, 1987*b*; Crescenzi *et al.*, 1988; Stetler-Stevenson *et al.*, 1988; Ngan *et al.*, 1989; Cotter *et al.*, 1990). The tumour marker can be detected with high sensitivity by using primers specific to the bcl-2/JH translocated gene sequences, and one tumour cell can be detected among $10^5$–$10^6$ normal cells (Crescenzi *et al.*, 1988; Stetler-Stevenson *et al.*, 1988).

Bone marrow involvement in NHL is common in certain subtypes at onset or during the course of the disease, and patients with bone marrow involvement run a substantial risk of tumour recurrence (Dick *et al.*, 1974; Rosenberg, 1975; Stein *et al.*, 1976; Simon *et al.*, 1988). Thus, improved assessment of occult lymphoma cells in this site, especially in early-stage NHL patients, could be very useful for staging and managing treatment of the disease. To this end, we have assessed the usefulness of bcl-2/JH PCR analysis on the peripheral blood and bone marrow of follicular lymphoma and intermediate-grade NHL patients.

### Material and methods

### *Patients and samples*

Blood and/or bone marrow samples to be analysed by PCR were collected from patients with non-Hodgkin's lymphoma (NHL) during the period from 1989 to 1991 by the department of medical oncology in Ticino, Switzerland. For this study, bone marrow samples were available from 71 patients: 33 were classified as follicular lymphoma, subtypes B, C and D, and 38 were classified as intermediate-grade NHLs, subtypes F and G according to the Working Formulation (WF) (The non-Hodgkin's lymphoma pathologic classification project, 1982). Mononuclear cells were separated by Ficoll-Hypaque centrifugation (Ficoll-Hypaque separating solution, Biochrom KG, Berlin) and red cells were removed with lysis buffer (0.32 M sucrose, 10 mM Tris-HCl, 5 mM $MgCl_2$, 1% Triton X-100) (Higuchi, 1989). The pellet containing mononuclear cells was resuspended ($5 \times 10^6$ cells per 1 ml) in 100 mM NaCl, 25 mM EDTA, 0.5% SDS and 200 $\mu g\ ml^{-1}$ proteinase K (Sigma, St Louis, MO) and incubated overnight at 37°C. DNA was extracted by phenol-chloroform and precipitated with ethanol. Eight samples with small numbers of cells were prepared by resuspension in digestion mixture (1 × PcR buffer, 0.25% Tween 20, 0.6 $\mu l$ of 10 $mg\ ml^{-1}$ proteinase K per 100 $\mu l$) at a concentration of $5 \times 10^5$ cell $ml^{-1}$ and incubated at 56°C for 2'h followed by a 95°C 20 min inactivation step to inhibit proteinase K (Kawasaki, 1990).

### *Polymerase chain reaction (PCR)*

One and a half $\mu g$ of sample DNA were added to a reaction mixture which contained 25 $\mu mol\ l^{-1}$ of each primer, 100 $\mu mol\ l^{-1}$ of each dNTP, 1.5 U Taq polymerase (Cetus, Emeraville, CA) 1 × PCR buffer (50 $mmol\ l^{-1}$ KCl, 10 $mmol\ l^{-1}$ Tris-Cl, 1.5 $mmol\ l^{-1}$ $MgCl_2$ in a 50 $\mu l$ total volume. The PCR was performed in a Perkin Elmer Cetus thermal cycler, using as primers the oligonucleotide: 5'-CTC GGA TCC AGT TGC TTT ACG TGG CCT GT-3' for the MBR or 5'-GAC TCC TTT ACG TGC TAC C-3' for the mcr and 5'-GGA AGC TTA CCT GAG GAG ACG GTG ACC-3' for the JH consensus region.

After an initial denaturation step at 94°C for 5 min, PCR for either MBR or mcr was performed under the following conditions: denaturation at 94°C 30 seconds, annealing at 56°C 30 seconds and polymerisation at 72°C 30 seconds for 40 cycles. For experiments detecting the MBR breakpoint, DNA from a NHL B-cell line $DoHH_2$ was used as the positive control (Kluin-Nelemans *et al.*, 1991). Four highly selected positive DNA samples from NHL patients served as positive controls for the mcr breakpoint. We closely followed the guidelines for preventing contamination proposed by Kwok & Higuchi (1989), and because of the extreme sensitivity of the PCR system, considerable effort was devoted to running numerous controls. We evaluated the specificity of the PCR assays by testing them against 42 DNA samples

Correspondence: F. Cavalli.
Received 23 July 1992; and in revised form 30 November 1992.

from leukaemic controls, healthy subjects and a group of patients with other diseases. All these samples were negative. Several reagent controls were run during each experiment. In addition to the positive controls, well characterised negative DNA samples were routinely included in each run. Samples negative for both breakpoints were checked for intactness of DNA by amplifying a 250 bp fragment of the P53 gene.

*Liquid hybridisation*

Liquid hybridisation (Ehrlich *et al.*, 1990) was performed in a total volume of 40 µl using 20 µl of PCR product, 4 µl of salt solution (1.5 M NaCl, 25 mM EDTA), and 15,000 cpm of $\gamma$-$^{32}$P-ATP labelled internal probe, 5'CAC AGA CCC ACC CAG AGC CCT CCT GCC CTC CTT CCC GCG GG-3' for the MBR and 5'-CTC GGA TCC AGT TGC TTT ACG TGG CCT GT-3' for the mcr. For hybridisation, samples were denatured at 98°C for 5 min and annealed at 72°C for 15 min. Twenty µl of the hybridised product was loaded on a 3% NuSieve/0.8% agarose gel containing 0.5 × TBE buffer. After electrophoresis the gel was dried down under low heat vacuum, and subsequently analysed by autoradiography. Exposure times were generally between 2 and 6 h.

### Results

*Sensitivity of the assay*

Serial dilution experiments using the DoHH$_2$ cell line demonstrated that tumour cells can be detected by PCR in a dilution of at least $10^{-4}$ on ethidium-bromide stained agarose gels. Liquid hybridisation increased this sensitivity up to at least $10^{-6}$.

*Bone marrow histology vs bone marrow PCR detection*

Bone marrow from 71 low- or intermediate-grade NHL patients was tested by PCR for mbr and mcr breakpoints. Samples negative for both t(14;18) breakpoints were further tested for intactness of DNA by amplifying a short segment of P53 gene. Thirty-three bone marrows were available from follicular NHL (WF B, C and D) patients and Table I summarises the results of PCR testing. Twelve of fourteen patients whose bone marrow was positive by histology were also PCR positive (86%). We found in 19 morphologically 'negative' bone marrows a further 11 patients (58%) who were clearly t(14;18) positive by PCR. Representative PCR products from four of the patients with morphologically negative bone marrows are shown in Figure 1. Table II shows that when the same group of 33 follicular lymphoma patients are staged using the Ann Arbor system, many of the early-clinical-stage patients manifest the t(14;18) marker in their bone marrow.

The PCR assay detected the t(14;18) in 13 (34%) of 38 patients with intermediate-grade NHLs (WF F and G). When bone marrow histology was negative PCR demonstrated neoplastic cells in approximately 28% of samples (Table III).

**Table I** Comparison of bone marrow histology *vs* PCR in 33 follicular lymphoma patients (WF: B,C,D)[a]

| *Histology* | *No. of patients* | *PCR positive* |
|---|---|---|
| Bone marrow positive | 14 | 12 (86%) |
| Bone marrow negative | 19 | 11 (58%) |
| Total | 33 | 23 (70%) |

[a]WF: Working Formulation.
B–follicular, predominantly small cleaved cells.
C–follicular, mixed, small cleaved and large cells.
D–follicular, predominantly large cells.

**Figure 1** PCR detection for t(14;18) mbr in bone marrows with negative histology from follicular lymphoma (FL) patients. Autoradiography of annealed products shows strong positive signals over 4 of 5 morphologically negative bone marrow samples (lane 2–6). Pos.: positive control (lane 1). Neg.: negative control (lane 7). Note variation in amplified product size when compared to each other and the positive control.

**Table III** Incidence of bone marrow involvement by PCR in 38 diffuse NHL patients (WF: F,G)[a]

| *Histology* | *No. of patients* | *PCR positive* |
|---|---|---|
| Bone marrow positive | 20 | 8 (40%) |
| Bone marrow negative | 18 | 5 (28%) |
| Total | 38 | 13 (34%) |

[a]WF: Working Formulation.
F–diffuse, mixed, small and large cells.
G–diffuse, large cells.

*Peripheral blood correlate*

We attempted to determine the concordance rate between the peripheral blood–a more accessible tissue– and the bone marrow PCR assay. Fifty-six paired peripheral blood and bone marrow samples were tested by PCR and the concordance was striking, with the peripheral blood samples from 55 patients uniformly showing the same result as the corresponding bone marrow. Although these findings suggest that

**Table II** Bone marrow PCR *vs* histology and clinical stage of 33 follicular lymphomas patients (WF: B,C,D)[a]

| *Clinical stage* | *No. of patients* | *PCR positive* | *BM histology positive* |
|---|---|---|---|
| I | 3 | 1 (33%) | 0 |
| II | 6 | 3 (50%) | 0 |
| III | 6 | 4 (67%) | 0 |
| IV | 18 | 15 (83%) | 14 (78%) |

[a]WF: Working Formulation.
B–follicular, predominantly small cleaved cells.
C–follicular, mixed, small cleaved and large cells.
D–follicular, predominantly large cells.

peripheral blood and bone marrow findings are closely correlated this may no longer be true after treatment. We observed three patients with FL in whom peripheral blood became t(14;18)-negative following chemotherapy (single agent chlorambucil) while the bone marrow remained clearly positive. These data are very preliminary and a large number of patients will be followed prospectively.

### Discussion

The utility of molecular monitoring in the assessment of lymphoma patients is still controversial: conflicting results have been published on the prognostic significance of bcl-2 protein expression and/or bcl-2 gene rearrangements (Yunis *et al.*, 1989; Price *et al.*, 1991; Pezzella *et al.*, 1992).

In this study we assessed the usefulness of bcl-2/JH PCR as a means for improving the detection of lymphoma cells in the bone marrow of follicular lymphoma and intermediate-grade NHL patients. Half of the early-stage FL patients with morphologically normal marrow had amplifiable breakpoints, and we found that the marrow compartments, even in stage I and II patients, frequently contained lymphoma cells when tested by PCR. Thus bcl-2/JH PCR assessment in early-stage FL can result in upstaging many patients and may be of considerable interest to the clinician.

The possibility of an easy detection of bcl-2/JH rearrangement may also be useful in diffuse large-cell or mixed-cell lymphomas where it may be associated with a relatively poor prognosis (Yunis *et al.*, 1989) and may indicate a morphological transformation from a FL to a more aggressive histologic subtype (Lee *et al.*, 1987a). In the intermediate-grade patients we found bcl-2/JH rearrangements in approximately 34% of bone marrow and peripheral blood samples. This finding is consistent with other studies reporting an overall frequency of approximately 20%–40% of the t(14;18) in this group of patients (Yunis *et al.*, 1982; Lee *et al.*, 1987a; Aisenberg *et al.*, 1988; Yunis *et al.*, 1989; Lambrechts *et al.*, 1992).

The most interesting finding in our study was the striking correlation between bcl-2/JH positivity in the peripheral blood and the corresponding bone marrow sample. Paired samples showed concordance in virtually all cases. We believe that our data accurately reflect the status of the tissue samples and are not a consequence of cross-contamination or false positive results, because in all cases where bone marrow DNA was negative, the corresponding peripheral blood sample contained no detectable specific PCR product. In addition, the migration patterns on agarose gel suggested that the results were not due to contamination because the size of the PCR products varied from patient to patient. This observation suggests that the peripheral blood–a readily accessible tissue–provides considerable ancillary information in early-stage follicular lymphoma patients. Peripheral blood may in fact provide nearly the same information as the bone marrow aspirate. Therefore, it may reduce the need for more invasive examinations. Similar results, with a lesser degree of concordance between bone marrow and peripheral blood analysis, have recently been published (Hickish *et al.*, 1991) indicating the PCR can be an effective means for clinical monitoring of low-grade lymphomas. However, the clinical significance of detecting minimal disease in bone marrow by PCR has still to be established and will require prolonged follow-up because of the natural history of FL with a median survival than can exceed 8 years in advanced stages. Serial samples from patients undergoing chemotherapy suggest that after treatment the peripheral blood and bone marrow may show divergent results and a larger number of patients will be followed prospectively to confirm this very preliminary finding whose biological significance is yet unclear. One possibility is that circulating cells with the t(14;18) have altered properties (e.g. loss of adhesion) modifying their clinical behaviour. The clinical relevance of the persistence of PCR-detectable neoplastic cells in the blood and in the bone marrow may also be different for the different types of lymphomas. Further studies on serial samples from patients are needed to determine the usefulness of bcl-2/JH PCR detection post-therapy. In fact, it has recently been shown that aggressive chemotherapy often fails to eradicate bcl-2/JH-positive cells from the marrow even when this becomes morphologically negative (Gribben *et al.*, 1991) and that circulating cells carrying t(14;18) may be found in some long remissions of advanced FL (Price *et al.*, 1991; Lambrechts *et al.*, 1992) but not in patients initially with truly localised disease (Price *et al.*, 1991). An accurate quantification of the PCR analysis would be very helpful for clarifying many issues, development of reliable procedures is ongoing.

We thank Dr F.E. Cotter (LRF, Institute of Child Health, London) for providing us the DoHH2 cell line and Dr A.D. Zelenetz (Sloan-Kettering Cancer Center, New York) for the mcr positive DNA samples.

This work was partially supported by the SCHWEIZERISCHE KREBSLIGA (Swiss League against Cancer) and grant No. 0011 of the Research Service of the Department of Veterans Affairs. E. Zucca was an ESMO fellow.

### References

AISENBERG, A.C., WILKES, B.M. & JACOBSON, J.O. (1988). The bcl-2 gene is rearranged in many diffused B-cell lymphomas. *Blood*, **71**, 969–972.

BAKHSHI, A., JENSEN, J.P., GOLDMAN, P., WRIGHT, J.J., MCBRIDE, O.W., EPSTEIN, A.L. & KORSMEYER, S.J. (1985). Cloning the chromosomal breakpoint of t(14;18) human lymphomas: clustering around JH on chromosome 14 and near a transcriptional unit on 18. *Cell*, **41**, 899–906.

CLEARY, M.L. & SKLAR, J. (1985). Nucleotide sequence of a t(14;18) chromosomal breakpoint in follicular lymphoma and demonstration of a breakpoint-cluster region near a transcriptionally active locus on chromosome 18. *Proc. Natl Acad. Sci. USA*, **82**, 7439–7443.

CLEARLY, M.L., SMITH, S.D. & SKLAR, J. (1986*a*). Cloning and structual analysis of cDNAs for bcl-2 and a hybrid bcl-2/immunoglobulin transcript resulting from the t(14;18) translocation. *Cell*, **47**, 19–28.

CLEARY, M.L., GALILI, N. & SKLAR, J.A (1986*b*). Detection of a second t(14;18) breakpoint cluster region in human follicular lymphomas. *J. Exp. Med.*, **164**, 315–320.

COTTER, F.E., PRICE, C., ZUCCA, E. & YOUNG, B.D. (1990). Direct sequence analysis of the 14q+ and 18q– chromosome junctions in follicular lymphoma. *Blood*, **76**, 131–135.

CRESCENZI, M., SETO, M., HERZIG, G.P., WEISS, P.D. GRIFFITH, R.C. & KORSMEYER, S.J. (1988). Thermostable DNA polymerase chain amplification of t(14;18) chromosome breakpoints and detection of minimal residual disease. *Proc. Natl Acad. Sci. USA*, **85**, 4869–4873.

DICK, F., BLOOMFIELD, C.D. & BRUNNING, R.D. (1974). Incidence, cytology and histopathology of non-Hodgkin's lymphomas in the bone marrow. *Cancer*, **33**, 1382–1398.

EHRLICH, G.D., GREENBERG, S. & ABBOTT, M.A. (1990). Detection of human T-cell lymphoma/leukemia viruses. In *PCR Protocols: a Guide to Methods and Applications*. Innish, M.A., Gelfand, D.H., Sninsky, J.J. & White, T.J. (eds), pp. 325–336. Academic Press: INC.

GRIBBEN, J.G., FREEDMAN, A.S., WOO, S.D., BLAKE, K., SHU, R.S., FREEMAN, G., LONGTINE, J.A., PINKUS, G.S. & NADLER, L.M. (1991). All advanced stage non-Hodgkin's lymphomas with a polymerase chain reaction amplifiable breakpoint of bcl-2 have residual cells containing the bcl-2 rearrangement at evaluation and after treatment. *Blood*, **73**, 3275–3280.

HICKISH, T.F., PURVIES, H., MANSI, J., SOUKOP, M. & CUNNINGHAM, D. (1991). Molecular monitoring of low grade non-Hodgkin's lymphoma by gene amplification. *Br. J. Cancer*, **64**, 1161–1163.

HIGUCHI, R. (1989). Simple and rapid preparation of samples for PCR. In *PCR Technology*. Erlich, H.A. (ed), pp. 31–38. Stockton: New York.

HOCKENBERY, D., NUNEZ, G., MILLIMAN, C., SCHREIBER, R.D. & KORSMEYER, S.J. (1991). Bcl-2 is an inner mitochondrial membrane protein that blocks programmed cell death. *Nature*, **348**, 334–336.

KAWASAKI, E.S. (1990). Sample preparation from blood, cells, and other fluids. In *PCR Protocols: a Guide to Methods and Applications*. Innish, M.A., Gelfand, D.H., Sninsky, J.J. & White, T.J. (eds), pp. 146–152. Academic Press: INC.

KLUIN-NELEMANS, H.C., LIMPENS, J., MEERABUX, J., BEVERSTOCK, G.C., JANSEN, J.H., DE JONG, D. & KLUIN, P.M. (1991). A new non-Hodgkin's B-cell line (DoHH2) with a chromosomal translocation t(14;18) (q32;q21). *Leukemia* **5**, 221–224.

KWOK, S. & HIGUCHI, R. (1989). Avoiding false positives with PCR (published erratum appears in Nature 1989, 339, 490). *Nature*, **339**, 237–238.

LAMBRECHTS, A.C. DE RUITER, P.E., DORSSERS, L.C.J. & VAN'T VEER, M.B. (1992). Detection of residual disease is translocation (14;18) positive non-Hodgkin's lymphoma, using the polymerase chain reaction: a comparison with conventional staging methods. *Leukemia*, **6**, 29–34.

LEE, M.-S., BLICK, M.B., PATHAK, S., TRUJILLO, J.M., BUTLER, J.J., KATZ, R.L., MCLAUGHLIN P., HAGEMEISTER, F.B., VELASQUEZ, W.S., GOODACRE, A., CORK, A., GUTTERMAN, J.U. & CABANILLAS, F. (1987*a*). The gene located at chromosome 18 band q21 is rearranged in uncultured diffuse lymphomas as well as follicular lymphomas. *Blood*, **70**, 90–95.

LEE, M.S., CHANG, K.S., CABANILLAS, F., FREIREICH, E.J., TRUJILLO, J.M. & STASS, S.A. (1987*b*). Detection of minimal residual cells carrying the t(14;18) by DNA sequence amplification. *Science*, **237**, 175–178.

McDONNELL, T.J., DEANE, N., PLATT, F.M., NUNEZ, G., JAEGER, U., MCKEARN, J.P. & KORSMEYER, S.J. (1989). Bcl-2-immunoglobulin transgenic mice demonstrate extended B-cell survival and follicular lymphoprofileration. *Cell*, **57**, 79–88.

NGAN, B.-Y., CHEN-LEVY, Z., WEISS, L.M., WARNKE, R.A. & CLEARY, M.L. (1988). Expression in non-Hodgkin's lymphoma of the bcl-2 protein associated with the t(14;18) chromosomal translocation. *N. Engl. J. Med.*, **318**, 1638–1644.

NGAN, B.-Y., NOURSE, J. & CLEARY, M.L. (1989). Detection of chromosomal translocation t(14;18) within the minor cluster region of bcl-2 by polymerase chain reaction and direct genomic sequencing of the enzymatically amplified DNA in follicular lymphomas. *Blood*, **73**, 1759–1762.

PEZZELLA, F., JONES, M., RALFKIER, E., ERSBOLL, J., GATTER, K.C. & MASON, D.Y. (1992). Evaluation of bcl-2 protein expression and 14;18 translocation as prognostic markers in follicular lymphoma. *Br. J. Cancer*, **65**, 87–89.

PRICE, C.G.A., MEERABUX, J., MURTAGH, S., COTTER, F.E., ROHATINER, A.Z.S., YOUNG, B.D. & LISTER, T.A. (1991). The significance of circulating cells carrying t(14;18) in long remission from follicular lymphoma. *J. Clin. Oncol.*, **9**, 1527–1532.

ROSENBERG, S.A. (1975). Bone marrow involvement in the non-Hodgkin's lymphomata. *Br. J. Cancer*, **31** (suppl.2): 261–264.

SIMON, R., DURRLEMAN, S., HOPPE, R.T., BONADONNA, G., BLOOMFIELD, C.D., RUDDERS, R.A., CHESON, B.D. & BERARD, C.W. (1988). The non-Hodgkin's lymphoma pathologic classification project. Long-term follow-up of 1155 patients with non-Hodgkin's lymphomas. *Ann. Intern. Med.*, **109**, 939–945.

STEIN, R.S., ULTMANN, J.E. BYRNE, G.E., MORAN, E.M., GOLOMB, H.M. & OETZEL, N. (1976). Bone marrow involvement in non-Hodgkin's lymphoma. Implications for staging and therapy. *Cancer*, **37**, 629–636.

STETLER-STEVENSON, M., RAFFELD, M., COHEN, P. & COSSMAN, J. (1988). Detection of occult follicular lymphoma by specific DNA amplification. *Blood*, **72**, 1822–1825.

THE HON-HODGKIN'S LYMPHOMA PATHOLOGIC CLASSIFICATION PROJECT. National Cancer Institute sponsored study of classifications of non-Hodgkin's lymphomas. Summary and description of a working formulation for clinical usage (1982). *Cancer*, **49**, 2112–2135.

TSUJIMOTO, Y., FINGER, L.R., YUNIS, J., NOWELL, P.C. & CROCE, C.M. (1984). Cloning of the chromosome breakpoint of neoplastic B cells with the t(14;18) chromosome translocation. *Science*, **226**, 1097–1099.

TSUJIMOTO, Y., COSSMAN, J., JAFFE, E. & CROCE, C.M. (1985). Involvement of the bcl-2 gene in human follicular lymphoma. *Science*, **228**, 1440–1443.

WEISS, L.M., WARNKE, R.A., SKLAR, J. & CLEARY, M.L. (1987). Molecular analysis of the t(14;18) chromosomal translocation in malignant lymphomas. *N. Engl. J. Med.*, **317**, 1185–1189.

YUNIS, J.J., OKEN, M.M., KAPLAN, M.E., ENSRUD, K.M., HOWE, R.R. & THEOLOGIDES, A. (1982). Distinctive chromosomal abnormalities in histologic subtypes of non-Hodgkin's lymphomas. *N. Engl. J. Med.*, **307**, 1231–1236.

YUNIS, J.J., MAYER, M.G., ARNESEN, M.A., AEPPLI, D.P., OKEN, M.M. & FRIZZERA, G. (1989). Bcl-2 and other genomic alterations in the prognosis of large-cell lymphoma. *N. Engl. J. Med.*, **320**, 1047–1054.